# Food Markets: A Motivation-Based Segmentation of Tourists

**DOI:** 10.3390/ijerph17072312

**Published:** 2020-03-30

**Authors:** Ana Mᵃ Castillo-Canalejo, Sandra Mᵃ Sánchez-Cañizares, Luna Santos-Roldán, Guzmán Antonio Muñoz-Fernández

**Affiliations:** Department of Statistics, Business Organization and Applied Economics, Faculty of Law and Business Administration, University of Cordoba, Puerta Nueva s/n, 14071 Cordoba, Spain; dt1casca@uco.es (A.M.C.-C.); sandra.sanchez@uco.es (S.M.S.-C.); luna.santos@uco.es (L.S.-R.)

**Keywords:** food markets, motivation, segmentation, tourists, satisfaction, loyalty

## Abstract

Food markets are becoming popular as new spaces for recreation, and this research aims to discover the motivations driving the tourists that visit these markets. Factorial analysis, cluster analysis and Student’s *t*-test were applied on 456 surveys from two food markets in Córdoba (Spain). Three motivational factors were obtained: Gastronomic experience and novelty; hedonism and leisure; and the relationship of the experience with work. Segmenting and analyzing the profile of the tourist may help economic agents develop new strategies for the tourism management of a destination and more accurate marketing and branding strategies that target specific customers with a niche message. The study could help develop products that align with tourists’ motives to increase satisfaction and loyalty.

## 1. Introduction

When travelling, people do not only eat food to satisfy their physical and physiological needs [1]. In an era of accelerated globalization, they wish to enjoy diverse rather than mono-cultural environments; thus, good gastronomy has turned into a need for modern society [2]. The food of a destination has the potential to improve the visitor’s experience, as it allows them to: Connect with the place, its culture, and its heritage [3]; be part of the tourist experience [4]; and contribute to building a concept that is greater than the initial object of tourism itself [5], for example, with heritage tourism. That is to say, the consumption of local food may contribute to the promotion of a tourism destination’s brand [6], because this food is recognized as being part of the local culture [7]. Therefore, the knowledge of the sensory dimensions of a tourist’s experience is relevant for the improvement of tourism destinations [8].

On the other hand, the search for differentiation in order to improve the tourism offer leads cities to adopt products based on unique experiences. This results in the emergence of new places that try to cover the needs of the experiential visitor seeking cultural, culinary or creative alternatives [9]. In this case, traditional food markets are a reflection of the local culture and the traditions of the inhabitants of an area [10]. Additionally, they are spaces full of history, and are becoming an excellent and attractive resource for visitors [11]. The food market visitors are interested in discovering local products [12] as a way to introduce themselves to the destination’s culture.

In cities, there is a tendency to make these supply markets into tourism attractions in historic centers [13]. On numerous occasions, especially in tourist attractions and commercial cities, these markets are transforming from places of purchase of local food products into spaces for enjoying and learning about local gastronomy. Due to this, new types of markets, known as food markets, are appearing. According to Mora [14], they may be classified into four types: (1) Renovated traditional markets that have a balance between old and new food stands aimed at tourists and food visitors who want to try food in the same place; (2) re-invented markets, transformed into gastronomical leisure spaces while also preserving the essence and distribution of traditional markets; (3) open-air markets where gastronomic products and diverse objects are sold to locals and tourists; and (4) corrupted markets which preserve the original building structures but only offer international fashion shops and restaurants.

This study aims to discover the motivations and factors which determine satisfaction, and the characteristics of tourists to these emerging food markets in the city of Córdoba (Spain), a destination recognized as a World Heritage Site. It aims to verify if this type of area may have variables of interest within the experiential offer in a cultural tourism city. Definitively, it intends to contribute to the scant existing literature regarding food markets and fill in the gaps in the existing knowledge on the tourist motivations of local markets in destinations and identify through factor–cluster analysis. The results allowed the discovery of how these food experiences affect the visitor, so a segmentation proposal may provide insights to be taken into account for the development of future destination management policies, related to promotional activities and destination branding development.

In order to achieve this goal, field work consisting of a survey was given to a sample of visitors at these food markets. After this introduction, there is a review of the scientific literature focused on the constructs which are the subject of this study. 

## 2. Literature Review

### 2.1. Visitor Motivation of Food Markets

Motivation-based segmentation is very popular due to the extent of information that can be obtained. It helps to evaluate the needs of the market and how to satisfy customers and enhance the services offered. Motivations are also measured to identify and segment types of visitors for the purpose of product development and market promotion [15]. Crompton and McKay [16] argued for the importance of understanding motivations by giving three reasons: (1) Understanding motivations would pave the way for creating better products and services, (2) satisfaction with experiences is intrinsically related to initial motives and the identification of visitor motivation is key for modeling the event to satisfy visitors, and (3) motives must be identified and prioritized first before a destination marketer can understand tourist decision-making processes. Iso-Ahola [17] also stated that motivation is one of the most important determining factors for leisure events. 

A significant number of studies focus on festival attendees’ motivation, level of satisfaction and intention to return [15,18,19,20,21,22,23,24,25,26]. In general, findings from the literature indicate that dimensions of motivation are similar across all festivals; however, the specific components and factors may vary depending on the festivals, events and market [26]. Uysal and Li [27] reviewed existing empirical research of festival and event motivation. They classified the most frequently mentioned dimensions of festival motivations as: Socialization, family togetherness, novelty, escape, cultural exploration, entertainment, and excitement. 

Gastronomy as a variable for tourist segmentation in a particular destination is common in food tourism literature, but there is a lack of studies about segmentation of food markets’ attendees [10]. Table 1 summarizes the main findings of the studies that focused on the motives of people who attend gastronomic festivals, events or markets. It can be observed that the only studies that investigated motivations to visit a gastronomic market are Crespi-Vallbona and Dimitrovski [10] and Dimitrovski and Crespi-Vallbona [28]. Their 2016 study segmented the tourists that visit the Boquería market in Barcelona based on four motivational factors: interacting with local producers and vendors, sensory appeal, local food experience and healthy eating concerns. The second study tested the direct effects of escape from routine, cultural experience, prestige, and food market involvement on satisfaction, and also the moderating role of food neophilia in this relationship. Escobar-López, Espinisa-Ortega, Vizcarra-Bordi, and Thomé-Ortiz [29] analyzed the characteristics of consumers of organic food, based on their motivations. The focus of the remaining studies is tourist motivation in a food, culinary or gastronomic festival or event.

### 2.2. Socio-Demographic Variables

Examining the socio-demographic characteristics of the tourists in any destination may help the economic agents involved develop business strategies to increase the number of tourists and their satisfaction level.

The analysis of how the socio-demographic variables influence the motivations for attending a tourist destination, festival, etc. is very common in scientific literature [18,20,33,34,35] and in the field of food tourism, where there are studies without significant differences in the segmentation based on visitors’ motivations [10,21]. Others, meanwhile, have discovered an association between the cluster and specific socio-demographic variables [30,31,36].

### 2.3. Development of Hypotheses

Satisfaction is conceived as a determining factor in the success of markets [37]. It has a direct effect on business results and is accepted as an indicator of future profits of a business [38]. Luo and Homburg [39]; Yeung, Ging, and Ennew [40]; and Anderson, Fornell, and Lehmann [41] concluded that customer satisfaction positively affects the profitability of a business.

Understanding which motives influence the satisfaction and expectations of the tourist is one of the most relevant areas of research in the tourism sector, given the impact that it has on the success of any destination. A high level of tourist satisfaction leads to positive future behavior, such as the intention to return to the destination, reduce the elasticity of the prices, reduce the transaction costs and, increase recommendations [42,43]. This shows the importance of the management of the destinations in directing the tourism offer and satisfying the visitors’ needs. In this sense, measuring loyalty, based on the intention of returning to a place and recommending it to others, is key for the consolidation of a destination [44].

Previous studies confirm the relationship between the motives for visiting a place with satisfaction and loyalty. Thus, Grappi and Montanari [45] conclude that when there is a greater hedonistic feeling, the level of satisfaction is higher. Akhoondnejad [46] establishes a significant relationship between the authenticity and quality of a cultural festival with the satisfaction and loyalty of the visitor. Tanford and Jung [47] positively relate the place’s environment, the sense of escape, socialization, authenticity, and the quality–price relationship with satisfaction and loyalty to the festivals. Lee et al. [22] indicate that visitor satisfaction is influenced by motives for attending a cultural event.

In relation to events, festivals, and food markets, Vesci and Botti [48] indicate that the quality of the food and drink and the service and hospitality strongly determine the intention to return. Mason and Paggiaro [49] conclude that “festivalscape” has a direct effect on the satisfaction of the participants in culinary events. Crespi-Vallbona and Dimitrovski [10] and Dimitrovski and Crespi-Vallbona [28] show significant differences in the satisfaction with and intention to return to the food market of La Boquería in Barcelona (Spain) in terms of the motivations of the visitors: Interaction with local producers and vendors, sensory appeal, local food experience, health concerns, escape from routine, cultural experience, prestige, food market involvement, and food neophilia.

Considering the results of the literature, the following hypotheses are proposed:

**H1.**  *There are different groups of tourists depending on their gastronomic motivations.*

**H2.**  *The degree of satisfaction achieved in the gastronomic market is conditioned by the gastronomic motivations of the tourist.*

**H3.**  *The expectations over the gastronomic market are conditioned by the gastronomic motivations of the tourist.*

**H4.**  *Tourists present significant differences in the recommendation and the will to visit the gastronomic market again depending on their motivations.*

**H5.**  *Tourists show differences in the perception of gastronomy and the characteristics of the market depending on their motivation.*

**H6.**  *Discovering the cuisine is a factor that contributes to and conditions the experience in a gastronomic market.*

## 3. Materials and Methods

The city of Córdoba (Spain) has two food markets which, following the philosophy and strategy of traditional supply markets, have created this new leisure resource for experiential visitors. The main idea of this type of market is to bring food directly to the plate from each stand, where the food offer is displayed. In this way they become small restaurants that serve plates in their own style. Thus, the customer can choose the products they want from a wide range of food.

These markets are Mercado Victoria and Los Patios de la Marquesa, which, according to Mora’s [14] previously described types, follow the approach of situating reinvented food markets in emblematic buildings. Mercado Victoria, located in the center of the city, is a wrought iron building dating from 1877 and has an approximate surface area of 2000 square meters. Nearly 30 stands offer local, fusion and international food here.

The second food market in the city is Patios de la Marquesa, which is located in the historic center of the Jewish Quarter (recognized as a World Heritage Site by UNESCO), very close to the Mosque-Cathedral. This market occupies an old manor house from the 18th century and its 17 stands cover more than 1000 square meters. In this market, there is a mixture of gastronomy, and art. They organize events such as exhibitions for photography, painting, fashion, or show cooking. They even have live flamenco performances.

This study uses data from a survey carried out on tourists to these two food markets from November 2018 to February 2019, on days of high attendance, usually evenings from Thursday to Saturday. The aim was to discover the profile of the visitor, their motivations for visiting and their assessments of the experience. The survey was carried out within the markets, while the tourists were enjoying their food. No stratification was done for any socio-demographic variable as there is not a previous profile of this type of tourist. A convenience sample, where those surveyed are interviewed in a specific space and time [50], was used. An interviewer addressed tourists requesting their collaboration to answer the questionnaire; if they agreed to participate, they received the document to be completed. The chosen tourists were both Spaniards and foreigners. The survey (Supplementary Materials) was distributed in two languages (Spanish and English) and only to tourists, not to local visitors (a control question was designed to assure that the survey was only answered by tourists). Prior to the definitive survey, a pre-test was carried out on 20 individuals in order to determine the validity of the questionnaire. The rejection rate in the definitive survey was low and insignificant. A total of 500 answers were obtained, of which 456 were used. The rest were rejected due to improper completion. 

The variables that they intended to measure are related to the analysis of motivation, satisfaction, loyalty, and socio-demographic characteristics. To elaborate the questionnaire, we relied on scientific literature dealing with the analysis of motivation, satisfaction, expectations and loyalty in the context of gastronomic festivals or gastronomic destinations [10,16,22,23,31,32,51,52]. The questionnaire has a structure that consists of four blocks. The first regards the interest, attitudes and motivations related to the visit and the consumption of foodstuffs in the food markets. A second block deals with the assessment of different aspects of the markets. A third block is about the expectations of the survey´s subjects, its general assessment and loyalty. All the questions included in these three blocks were assessed according to a 5-point Likert scale with 5 being the highest score in all of the cases. Finally, the fourth block analyses the socio-demographic characteristics of the survey´s subjects.

The reliability index according to Cronbach’s alpha coefficient was analyzed to show the internal consistency of items when appropriate. Results highlight an acceptable index to reinforce the validity of the research work carried out [53].

## 4. Results and Discussion

The chosen sample (Table 2) comprises mainly youths (73.0%) who are under the age of 30, with women predominating. The education level is high as more than 85% hold a university degree. The tourists to the two food markets are overwhelmingly Spaniards. In terms of the type of attendance in these markets, more than 54% have visited this kind of establishment for the first or second time.

These percentages do not show significant differences if they are analyzed separately in each of the two markets studied. Only the percentage of previous visits is higher in Patios de la Marquesa (51.2% for 2–3 times compared to 36.4% in Mercado Victoria).

The questionnaire included ten possible motivations for visiting the food markets, which the respondents pondered on. According to the average assessment obtained (Table 3), the most important reasons focused on enjoying time with friends or family, followed by relaxing and disconnecting from the daily routine, and the fame and good reputation of the market. On the other hand, the aspects related to work (doing business, building contacts, and networking) have barely been assessed. No significant differences were found in the motivations of tourists in the two markets studied. Only the reason referred to as “doing business or work” has a significantly higher average in Mercado Victoria, this market is nearer to the city´s CBD (central business district), hence the common answer.

The segmentation of tourists has been used in gastronomic tourism as an instrument to analyze different researches. In this case, aiming to reduce the number of motivational variables, we proceeded to do an exploratory factor analysis with extraction by principal components and varimax rotation, where three factors have been obtained (Table 4) with eigenvalues higher than 0.5 [54] which explain a total of 58.403% of the variance. The Kaiser–Mayer–Olkin measure equals 0.794 and the Bartlett test of sphericity is significant. 

The 10 items from the motivations were reduced to three factors. Factor 1, which has been named food experience and novelty, has the highest variance percentage, almost 25%, and brings five related items together. These are the search for new experiences in food and drink, tasting dishes or favorite foods, the desire to try new things, relaxation and disconnection from daily life, and the good reputation of the market.

Factor 2 is hedonism and leisure, which explains more than 18% of the total variance. It includes three items related to the enjoyment of time with relatives and friends, being a leisure activity and a good quality–price relationship.

Finally, Factor 3 is known as relationship of the experience with work, which explains a reduced percentage of the variance (15.081%) and only includes two items focused on the use of time in the market for less formal work meetings or to develop networking opportunities.

Given that the factor analysis is statistically appropriate, the three factors obtained have been used as variables to perform a k-means cluster analysis in order to obtain two differentiated groups in the sample of tourists to food markets.

The first cluster is formed by 201 individuals (44.08% of the sample) and the second one by 244 visitors (the remaining 53.51%); there are 11 lost values (no answer). We analyzed the way that the motivations of each one of the three obtained factors were differentiated between the two clusters (Table 5). By doing so, it is observed that the second group assesses slightly better the motivations related with food experience, this difference is significant in all the surveyed motivations except for “new eating and drinking experiences” and “desire to do new things”. It also valued better the motivations related with hedonism and leisure. However, Cluster 1 always significantly assessed the aspects related to the Relationship of the experience with work above the second group. These results show that gastronomic motivations of tourists visiting the food markets are heterogeneous and, therefore, they can be segmented according to their culinary motivations (H1). Hence, we can say that besides the motivations related with food experience in this kind of places, visitors have other motivational factors associated like hedonic and/or factors related to work relations. This last motivational factor is, as we highlighted before, more present in food markets closer to the CBD such as Mercado Victoria. 

After the segmentation was done, we analyzed the possible existing relationships between each of the segments obtained with the four factors that are relevant in the analysis of the visit: Satisfaction, expectations, recommendation to others, and intention to return.

Specifically, a Student’s *t*-test was used for independent samples to contrast how different the means are (Table 6), which reflects the rejection of the null hypothesis in all the cases for a confidence level of 99%, except for the expectations (hypothesis H3 is rejected). This confirms that the differences in the averages of both groups in terms of satisfaction (H2), recommendation, and intention to visit a place again (H4), are significant. This shows enough robustness for the statistical analysis in terms of Type I errors, before possible violations of the normality for this type of distribution [55]. In each one of the four variables, it can be seen how the second cluster—the most numerous—most positively assesses all these aspects. In both groups, the intention to visit the market again has the highest average score. These four aspects also show significant differences depending on the market visited by tourists, always being statistically higher in Patios de la Marquesa than in Mercado Victoria.

It can be confirmed that the visitor of a food market who has a greater motivation related to culinary and hedonic aspects will be a visitor who will have a greater satisfaction in general. This visitor will also have a greater chance of recommending this food market to family and/or friends when he or she returns from the trip, and, if the visitor returned to the destination, he will visit the food market again, highlighting its loyalty towards it [48].

The preferences of the visitors regarding a series of products offered in the food markets (seafood, fish, meat, cold meats, desserts, wines and traditional dishes from the city) was also analyzed. On this occasion, the analysis of the difference of means between the two clusters was not significant, except in the case of traditional dishes from the city, which are preferred to a greater extent by group 2 (average 4.26 compared to 4.00 in group 1). These traditional dishes are the favorites for both clusters. Another parallel is that both clusters indicate seafood as the product they like least.

Additionally, the importance the tourist considers the traditional food of the city to have for its image as a tourist destination has been assessed, along with a series of elements which characterize the market they have visited. Table 7 reflects the average scores of each group, as well as the Student’s *t*-test to contrast how similar the means are. 

Although for both group, the traditional gastronomy of the city holds great importance in their tourist image, it is Group 2 which assesses this aspect with a higher score. Moreover, all the analyzed characteristics regarding the food market are assessed higher by Cluster 2 with statistically different means (hypothesis H5 is accepted), with the exception of prices, and the innovation and new flavors of the dishes offered. In any case, both groups coincide by indicating that the prices of these establishments are the aspect which they perceive to be the worst.

On the other hand, both the group of 244 tourists as well as that of 201 indicated atmosphere as the most highly regarded element, followed in this order by traditional gastronomy, facilities, and service and hospitality. These average perceptions do not show significant differences between the tourists that visit both markets. Only the average of "facilities" and "atmosphere" is statistically higher in Patios de la Marquesa.

To conclude with the analysis of clusters, the possible socio-demographic differences among the two clusters as well as the relationship between the market visited and the cluster it belongs to have been verified. Using contingency tables, the nonexistence of an association between the cluster and the academic training of the tourist has been verified. However, the Chi-squared or Cramer’s V coefficients for the nominal x nominal case reflect the existence of a significant association for the remaining characteristics. 

It is shown that the number of women is higher in Cluster 2 in comparison to Cluster 1 (165 to 114), with the opposite being the case for men (87 in Group 1 and 79 in Group 2). In addition, nationality reflects significant differences and, although the Spanish tourists dominate both groups when compared to the foreigners, the latter are found to be better represented in Cluster 1 (23 to 16) with there being more Spaniards in the second group (228 to 178). This result shows that foreign tourists evaluate most of the aspects lower than Spaniards, in referring to motivations such as satisfaction, expectations, recommendations or intention to return, except for the gastronomic experience, which is their main motivation. The result is logical in that this type of tourist will show a higher interest in discovering the traditional dishes and drinks of the city where they are staying as tourists. On the other hand, the Spanish tourists are likely to have already discovered these dishes. We can claim that discovering the gastronomy contributes and conditions to the experiences in food markets (hypothesis H6 is accepted).

Therefore, the experience of leisure and hedonism form a higher motivation factor for them.

In terms of age, in all the levels over 30 years of age, more cases are found in the first cluster. However, those under 30 years of age are found more often in the second (195 to 137).

The differences in the two groups regarding the food market they visited are also statistically significant. While the Mercado Victoria is represented more in Cluster 1 compared to Cluster 2 (90 tourists compared to 81), Los Patios de la Marquesa has more tourists in Group 2 than in 1.

Finally, the frequency of the visit also shows clearly differentiated results per cluster. In Group 1, those who visit the market for the first or second time (137 cases) predominate, while in Cluster 2 there is an evident majority of individuals who have visited the market more than twice (143 compared to 64 from the first group).

## 5. Conclusions

In tourist and business cities alike, new types of markets known as food markets are emerging. Their aim is to provide different options to the tourist who is seeking news culinary, creative, and cultural experiences. Given the scant literature in this field, this analysis may be of great use in understanding how the motivations of visitors may affect the perception of the satisfaction, expectation and loyalty towards the food market. Knowing the type of tourist, also aims to help the economic agents involved in this sector develop appropriate strategies which will result in greater benefits for tourist destinations.

This study analyzed the tourist segmentation of the two food markets in the city of Córdoba (Mercado La Victoria and Patios La Marquesa). The research concludes that tourist show three primary motivational factors: Gastronomic experience and novelty, hedonism and leisure, and the relationship of the experience with work. Taking into account these three factors, they also showed two clusters with significant differences in motivational factors, satisfaction and loyalty, with regards to the perceptions of different aspects of the food market and of the socio-demographic variables. 

The first group of tourists, which is smaller, visits the market mainly for new eating and drinking experiences, the desire to do new things, and to spend time with relatives and friends. It has a greater number of foreigners and men over the age of 30. The levels of satisfaction, loyalty and assessment of the market aspects are lower in this group. The second, larger cluster rates the majority of the options related to experience, gastronomic novelty, and hedonism and leisure more than the first group. They mainly visit the market for reasons related to the fame and reputation of the market, relaxation and disconnection from daily life, and to spend time with relatives and friends. In it there are more Spaniards, women, and people under the age of 30. The assessments of satisfaction, loyalty, and market aspects is higher. The reasons related to the experience-work relationship are the lowest in both groups, although the first group values it significantly higher than the second group.

One of this research´s main contributions is identifying that satisfaction and loyalty are mainly conditioned by the culinary motivations. Furthermore, hedonism and leisure motivations also contribute to the gastronomic satisfaction and loyalty towards the food markets. Hence, we conclude that these markets cannot focus only on food, they have to offer recreational activities that ease the hedonism and leisure of the visitor. 

This research not only has theoretical implication but also practical ones, the segmentation, done in this paper, may help manage these types of markets and develop marketing strategies and products that are more in line with the motives of their customers and their socio-demographic characteristics. Foreign tourists, as expected, show a greater interest in new experiences of eating and drinking, and Spaniards are more motivated by the experience of relaxation, disconnection and the opportunity to spend time with relatives and friends at the food markets. Given that this group is more satisfied and proves to be more loyal, it would perhaps be recommended to improve the aspect related to the motives of the experience-work relationship at a food market. It would also be interesting to reconsider the prices of the products on offer, with the price being one of the worst rated aspects for both groups.

As can be observed in this study the foreign tourists represent a low percentage, so it will be interesting to promote internationally this type of gastronomic tourism that is not good known yet and have a great potential.

The biggest limitation of this work is the survey’s methodology and the geographical area of the study performed, which makes the generalization of the results difficult. Future research could include other variables such as the amount of money spent, the number of establishments visited, other motives for the visit related to health, natural products, prestige. It could also make comparisons with other food markets in different cities and countries.

## Figures and Tables

**Table 1:** Motivation dimensions of gastronomic markets, events and festivals.

Author(s)	Study Focus	Dimensions of Motivation
[23]	What major factors attracted tourists to attend the South Wine and Food Festival in Miami Beach, Florida	Taste new wine and food Enjoy the event Enhance social status Escape from routine life Meet new people Meeting the celebrity and wine experts Spend time with family
[30]	Segmenting visitors to a culinary event based on motivations, travel behavior and expenditures	Food event Event novelty Socialization
[31]	Investigate visitors’ motivation for attending food festivals	Wine Escape and Event Novelty Food Known-group socialization External socialization Art
[10]	Motivations for attending food festivals	Interacting with local producers and vendors Sensory appeal Local food experience Health concern
[28]	Differentiate clusters of tourists on food markets	Escape from routine Cultural experience Prestige Food market-involvement Food neophilia
[32]	The study of local food markets and visitors’ motivation and satisfaction	New food experience Culture Socialization
[29]	The segmentation of the tourists who visit a gastronomic festival in the city of Guayaquil, Ecuador	Ecological concern Nutritional concern Availability of natural product Sensory aspects Health and confidence Economic aspects

Source: own development.

**Table 2:** Socio-demographic profile.

Gender	N (%)	Educational Background	N (%)
Male	168 (36.8%)	Elementary/primary school or less	18 (3.9%)
Female	288 (63.2%)	Vocational education /secondary school	47 (10.3%)
		Higher vocational diploma, bachelor’s degree	324 (71.1%)
Age		Master’s degree or PhD	67 (14.7%)
<30 years	333 (73.0%)		
30–39 years	41 (9.0%)	Times in gastronomy market before	
40–49 years	38 (8.3%)	1–2 visits	248 (54.4%)
50–60 years	39 (8.6%)	2–3 times	208 (45.6%)
Over 60 years	5 (1.1%)		
Nationality			
National	417 (91.5%)		
Foreigner	39 (8.5%)		

**Table 3:** Motive for the visit to the food market.

Motivation	Average	Standard Deviation
Eat and drink my favorite food/drink	3.490	1.163
New eating/drinking experiences	3.603	1.183
Fame and reputation of the market	3.770	1.092
Do business or work	2.048	1.286
Spend time with relatives, friends, colleagues	4.320	0.920
Being a leisure option near my home	3.330	1.450
Good quality/price relationship	3.467	1.029
Relaxation, disconnection from daily life	3.904	1.035
Desire to do new things	3.641	1.178
Networking	2.456	1.421

**Table 4:** Factor analysis.

Motivation	Factor 1	Factor 2	Factor 3
New eating/drinking experiences	0.755		
Fame and reputation of the market	0.562		
Relaxation, disconnection from daily life	0.604		
Desire to do new things	0.761		
Eat/drink my favorite drink/food	0.581		
Spend time with relatives, friends, colleagues		0.721	
Being a leisure option near my home		0.765	
Good quality/price relationship		0.559	
Do business or work			0.880
Networking			0.666
% variance explained	24.829	18.492	15.081
Cronbach’s Alpha	0.754	0.765	0.707

**Table 5:** Analysis of differences in motivation per cluster.

**Factor 1**	**Cluster 1**	**Cluster 2**	**Student *t*-Test**
New eating/drinking experiences	3.6269	3.6107	0.145
Fame and reputation of the market	3.5025	3.9754	−4.609 **
Relaxation, disconnection from daily life	3.4975	4.2418	−8.015 **
Desire to do new things	3.6418	3.6557	−0.124
Eat/drink my favorite food/drink	3.3532	3.6475	−2.703 **
**Factor 2**			
Spend time with relatives, friends, colleagues	3.7512	4.8074	−14.833 **
Being a leisure option close to my house	2.9204	3.5451	−4.653 **
Good quality/price relationship	3.2040	3.6639	−4.738 **
**Factor 3**			
Do business or work	2.6468	1.5902	9.384 **
Networking	2.9154	2.0779	6.406 **

** *p* < 0.01.

**Table 6:** Differences per cluster in satisfaction–expectations–recommendation-return.

Variables	Global Average	Std. Deviation	Cluster 1 (201 Cases)	Cluster 2 (244 Cases)	Student *t*-Test
Satisfaction	4.0548	0.7347	3.9254	4.1639	−3.376 **
Expectations	4.0175	0.7014	3.9403	4.0738	−1.993
Recommendation	4.2456	0.8367	4.0498	4.4180	−4.627 **
Return	4.3246	0.8173	4.1194	4.5000	−4.899 **

Cronbach’s Alpha = 0.830; ** *p* < 0.01.

**Table 7:** Differences by cluster regarding the perception of the gastronomy and the characteristics of the food market visited.

Item	Global Average	Std. Deviation	Cluster 1	Cluster 2	Student *t*-Test
Importance of traditional gastronomy for the tourist image	4.6491	0.5504	4.5821	4.7213	−2.699 **
Quality of dishes	3.7664	0.9010	3.6032	3.8904	−3.257 **
Prices	3.2740	1.0339	3.2462	3.2845	−0.380
Facilities	3.8750	0.9206	3.7487	3.9826	−2.593 **
Atmosphere of the establishments	4.0667	0.9272	3.8586	4.2232	−4.025 **
Innovation and new flavors in the dishes	3.5477	1.0162	3.5054	3.5767	−0.696
Service and hospitality	3.7926	0.9186	3.6875	3.8745	−2.086 *
Traditional gastronomy	3.9697	0.9675	3.7865	4.1327	−3.690 **

** *p* < 0.01, * *p* < 0.05.

## Supplementary Materials

The following are available online at https://www.mdpi.com/1660-4601/17/7/2312/s1.
