# Supplementary material for: Food Markets: A Motivation-Based Segmentation of Tourists
Source: Int J Environ Res Public Health. 2020 Mar 30;17(7):2312. doi: 10.3390/ijerph17072312 (PMC7178125; doi:10.3390/ijerph17072312)
Supplement: Supplementary file 1 [file ijerph-17-02312-s001.pdf]

UNIVERSIDAD DE CORDOBA

GASTRONOMIC TOURISM IN THE CITY OF CORDOBA

The University of Cordoba is carrying out research to find out the perception and opinion of visitors to the city's gastronomic markets. We ask for your cooperation and ask you to answer sincerely the following questions (it will take 4-5 minutes). If you have any doubts about any of the questions, please contact the interviewer. Thank you for your cooperation.

1) Sex:

1 ❑ Male 2 ❑ Female

2) Age:

1 ❑ < 30 years
2 ❑ 30-39 years
3 ❑ 40-49 years
4 ❑ 50-60 years
5 ❑ over 60 years

3) Educational background (mark with an X)

1		Elementary/Primary school or less
2		Vocational education or secondary school
3		Higher vocational diploma, Bachelor's degree
4		Master's degree or PhD

4) City and country of origin:

City:____________________ Country:_______________________

5) Your net monthly income:

1 ❑ less than 500 EUR
2 ❑ from 500 to 1.000 EUR
3 ❑ from 1.001 to 1.500 EUR
4 ❑ from 1.501 to 2.000 EUR
5 ❑ from 2.001 to 2.500 EUR
6 ❑ from 2.501 to 3.000 EUR
7 ❑ more than 3.000 EUR

6) How many times have you been in a gastronomy market in Córdoba before?

1 ❑ This is my first visit
2 ❑ I have been two or three times
3 ❑ I am a regular visitor (four times or more)

7) Indicate your preferences or tastes regarding the dishes/drinks offered in the market (1: nothing to 5:much)

	Grade				
Seafood	1	2	3	4	5
Fish	1	2	3	4	5
Meat	1	2	3	4	5
Cold meats	1	2	3	4	5
Desserts	1	2	3	4	5
Wines	1	2	3	4	5
Typical dishes of Cordoba	1	2	3	4	5

8) Grade the importance of the motives for visiting this place (1 – not important at all, 5 – very important)

	Grade				
Eating and drinking my favourite food/drink	1	2	3	4	5
New eating/drinking experiences	1	2	3	4	5
Fame and reputation of the market	1	2	3	4	5
Doing business or work	1	2	3	4	5
Spending time with relatives, friends, colleagues	1	2	3	4	5
Being a leisure option near my residence	1	2	3	4	5
Good quality/price	1	2	3	4	5
Relaxation, disconnect from daily life	1	2	3	4	5
The desire to do new things	1	2	3	4	5
Other	1	2	3	4	5

9) How important do you think is typical gastronomy for the tourism image of Córdoba (1 – not important at all, 5 – very important)

1	2	3	4	5

10) Rank from 1 to 5 your satisfaction with the following points of the market.

	1 (very poor)	2	3	4	5 (very good)	I don't know
Quality of the dishes						
Prices						
Facilities						
Atmosphere in the establishments						
Innovation and new tastes in the dishes						
Service and hospitality						
Traditional gastronomy						

11) Can you rank, from 1 to 5, your general level of satisfaction with the market? (Mark with an X)

Very unsatisfied				Very satisfied
1	2	3	4	5

12) 12. In a general way, what were your expectations regarding the quality of the services you expect to receive in the market? (Mark with an X)

Very low				Very high
1	2	3	4	5

13) Would you recommend this market if someone asked your advice? (Mark with an X)

Not at all				For sure
1	2	3	4	5

14) After your experience, do you think visit this market again? (Mark with an X)

Not at all				For sure
1	2	3	4	5

Thank you very much for your collaboration
